# Facing COVID-19 Challenges: 1st-Year Students’ Experience with the Romanian Hybrid Higher Educational System

**DOI:** 10.3390/ijerph18063058

**Published:** 2021-03-16

**Authors:** Sabina Potra, Adrian Pugna, Mădălin-Dorin Pop, Romeo Negrea, Luisa Dungan

**Affiliations:** 1 Management Department, Politehnica University of Timisoara, 300223 Timisoara, Romania; sabina.potra@upt.ro (S.P.); adrian.pugna@upt.ro (A.P.); 2 Research Centre in Engineering and Management, Politehnica University Timisoara, 300223 Timisoara, Romania; 3 Computer and Information Technology Department, Politehnica University of Timisoara, 300223 Timisoara, Romania; 4 Mathematics Department, Politehnica University of Timisoara, 300006 Timisoara, Romania; romeo.negrea@upt.ro; 5 Mechanical Machines, Equipment and Transports Department, Politehnica University of Timisoara, 300222 Timisoara, Romania; luisa.dungan@upt.ro

**Keywords:** grounded theory, face-to-face education, online education, hybrid system, student motivation, online asynchronous interviews

## Abstract

First-year students undergo several transformations like the transition from high school to university schedules, teaching methods, and discipline specificities to social changes that come with meeting new colleagues or moving to other locations far from family and friends. The COVID-19 outbreak brought additional concerns and uncertainties once educational systems implemented distance learning schemes for public health management. Nevertheless, higher educational organizations preferred to implement hybrid approaches for student engagement motivation and high dropout rate avoidance. In Romania, such an educational system has been applied with caution. Since the pandemic needs to be understood as an opportunity for adaptation and education improvements, the purpose of the present paper is to uncover lessons learned and to develop a systematized model based on students’ perception regarding face-to-face, online and hybrid systems. For this reason, a grounded theory approach has been preferred. Thus, 149 Romanian students enrolled in the first year in engineering specialities with ages between 18–26 years both male (50.3%) and female (49.7%) have answered the research questions in an online environment due to pandemic restrictions. Based on the online asynchronous student interviews, 220 codes and a further 13 categories have been developed. After a pertinent analysis of the relationships between categories and relevant literature sorting, a theoretical model for the Romanian higher educational current pandemic situation has been proposed. The main face-to-face and online education characteristics are outlined, the hybrid approach becoming a bridge between the two systems. Management implications are outlined together with further research directions.

## 1. Introduction

Throughout the last years, scholars and practitioners alike have been concerned about high dropout rates in higher education institutions, especially in engineering degrees [1,2,3]. The factors associated with this situation differ from low levels of prior qualifications [4], psychological distress [5] to the interaction with colleagues and institutional characteristics [6]. First-year students experience many changes in a very short period like shifts in socialization ties or educational requirements.

With the outbreak of the COVID-19 pandemic, changes happened instantly, and the higher education environment was no exception. Universities stopped face-to-face education and have been advised to rapidly offer online learning tools [7]. University staff and educators had to meet new online education challenges: demonstrating pedagogical skills in an online classroom, addressing their managerial role, establishing relationships with students, and providing technical support [8].

Distance education and online courses have long experienced high dropout rates [9]. Isolation and disconnectedness may lead to a loss of motivation to learn since the interaction is one of the most important factors in student satisfaction [10]. This adjustment to a new public health problem—the online exclusive education system implementation with no prior experience in most cases had an additional negative impact on first-year students’ engagement motivation.

Romania suspended classes at the beginning of March 2020 and started online teaching on specific educational platforms and with the help of video conferencing tools. All these shifts together with the need for fast adaptation determined serious gaps and improvised teaching methods. The second semester has been overcome and students hoped for a new university year with face-to-face classical learning. After the first COVID-19 wave, in September 2020 universities had to decide their strategy in fluctuating government scenarios. Politehnica University Timisoara chose to start the first five weeks exclusively online for older students and master students and a hybrid system for the first year students in several of its faculties [11]. The main reason for this decision resided in the fact that drop-out rates in first-year students are very high and they need face-to-face interaction to know each other and their teachers, to make bounds and to internalize their belonging to their specialization.

The hybrid approach combined face-to-face interactions in seminars and laboratories with online courses because it is defined by a substantial portion of online and a substantial portion of face-to-face education as argued by [12]. For the first four weeks, first-year students came to their faculties respecting distance restrictions, were organized in small groups, and asked to use protective masks throughout the educational activities. After the set time, due to pandemic high infection rates, students continued their classes exclusively online.

Their experience with three different educational systems in a very short period is very valuable for future educational improvements. Research on student perceptions in the face-to-face and online environment is well documented [13,14,15] and we will probably witness an increased interest in this topic in the following years due to the pandemic limitations and adaptation requirements. Even if students favour face-to-face education/onsite courses [12,13], their perception of online education can improve with online experience [12].

Therefore, the present research has chosen a grounded theory approach with the final purpose to determine face-to-face, hybrid and online characteristics based on Romanian first-year students’ perception in a technical university. In the methodology section, the research steps are outlined together with the details related to the sample size. The interviews data processing and results chapter synthesizes the main outcomes of the research. Based on literature sorting, the final model is proposed, and the conclusions are drawn.

## 2. Methodology and Methods

Grounded Theory (GT) is a theory-building qualitative research approach with high importance for management theory and practice [16]. It has sociological origins, being a method that discovers theoretical implications from data [17]. GT is widely used for qualitative data analysis [18,19,20] being systematic and at the same time flexible enough to collect and analyse data for an abstract conceptualization of people experiences [16]. Since it is best used in the absence of hypotheses [21], this approach has been considered appropriate in the present situation when we do not have previous theories regarding the hybrid education system.

**Figure 1:**
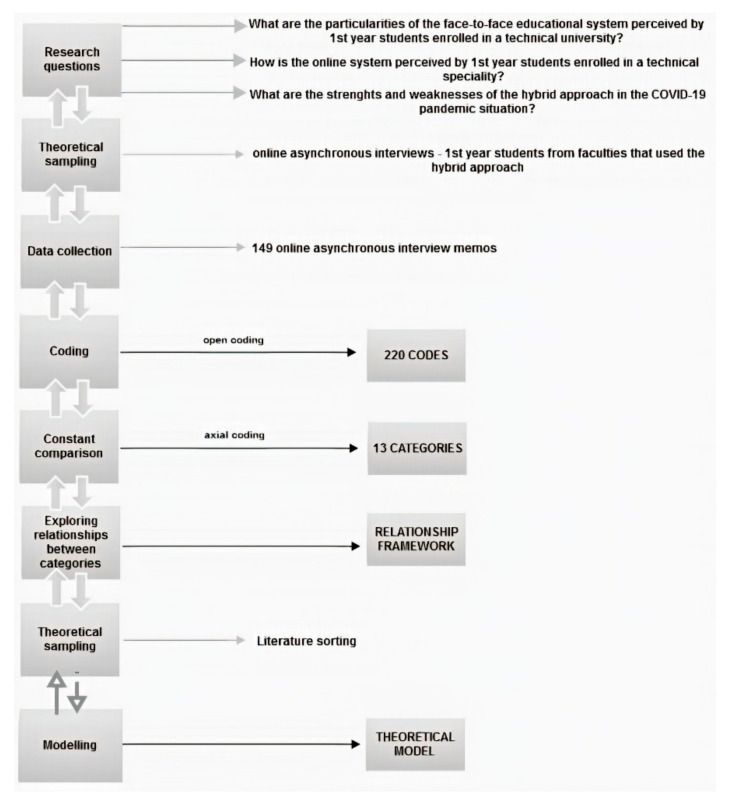
Grounded Theory steps for theoretical modelling.

The methodology steps are delineated in Figure 1. Regarding student perceptions in different educational systems, two streams of literature emerged: studies that concentrate on student characteristics or program features [15]. The present research envisages both, with an emphasis on the latter. Student characteristics taken into consideration are year of study, gender, technical speciality, and program characteristics largely explore all relevant features for the Romanian context. Scholars have identified components essential in student learning like course content, instructor teaching skills and course delivery modalities [13]. Since for the technical higher education institutions, we do not have prior prioritized learning components nor course delivery modalities, the article focuses on undercovering these particularities by delineating the following research questions:

1. What are the particularities of the face-to-face educational system perceived by 1st-year students in a technical university?
2. How is the online system perceived by these students?
3. What are the strengths and weaknesses of the hybrid approach in the COVID-19 pandemic situation?

However, we can acquire adequate data from students that are willing to discuss their experiences with this new system. Because some are shy or cannot express themselves when talking as they do in writing [22], and due to the COVID-19 restrictions, the online asynchronous interview technique has been chosen for the present research. The richness and quality of the data obtained via asynchronous interviews are considered very similar to that in face-to-face interviews [23]. Also, its benefits, like reduced time, easy transcription, multiple participants interviewed at a time, explicit and structured response nature due to enough time to think and formulate ideas, make this technique appropriate for this research.

Starting from the three research questions, the theoretical sampling step envisages decisions about which data to collect and how to do that. The online asynchronous interview technique has been preferred because it offers the only feasible way to collect data in the pandemic situation Romania encounters itself now. A semi-structured interview guide has been built with 16 questions ranging from personal questions regarding sex, faculty, and speciality, to informative general questions about initial faculty-related expectations and specific questions that explored the three educational systems’ characteristics related to interaction desire, platform accessibility, usability, attractiveness, assistance, and motivation. First-year students enrolled in Politehnica University Timisoara faculties that opted for face-to-face teaching seminars in the first month of the first semester 2020–2021 have been requested to answer online to the interview questions provided in the Appendix A. The interview guaranteed anonymity for all respondents.

Participants have been initially informed about the research subject, importance, and objectives through Zoom videoconferencing and Facebook groups. The interviewers were open to suggestions and pre-interview discussions emerged. Reminders have been used to raise the response rate.

For the sample size, an optimal stratified survey has been used. The general population was divided into sub-populations we call layers. The total number of population units is given in Equation (1).

(1) $$N_{1}+N_{2}+\ldots +N_{k}=\sum_{i=1}^{k}N_{i}=N$$

For each layer, we have *n_k_* random drawings with respect to Equation (2). The stratified survey is optimal if the *n_j_* level is dimensioned at a maximum efficiency level.

(2) $$n_{1}+n_{2}+\ldots +n_{k}=\sum_{i=1}^{k}n_{i}=n$$

We have established our sample size on linear characteristics [24]. The final sample size “*n*” is obtained from Equation (3) and the *n*_j_ values from Equation (4).

(3) $$n≅\frac{N\times z_{1-\alpha }^{2}\times \sigma_{b}^{2}}{N\times \delta^{2}+z_{1-\alpha }^{2}\times \sigma_{b}^{2}}$$

where $z_{1-\alpha }$ represents Laplace’s variable values, for a probability 1−α, where α is the significance level. In our case, *α* = 0.1 × (1 − *α* = 94%), thus $z_{0.1}=1.28$; $\sigma_{b}^{2}$- is the binary characteristic’s variance, that is $\sigma_{b}^{2}=p\times \left(1-p\right)$. To reduce statistical uncertainty, we have used maximum variance and the sample was designed as the worst-case scenario, that is $\sigma_{b}^{2}=0.5\times \left(1-0.5\right)=0.25$; $\delta^{2}$, the probable error. In our case, the average error is at a level of 6.8%.

(4) $$n_{j}=\frac{n\times N_{j}{\hat{\sigma }}_{j}}{\sum_{j=1}^{k}N_{j}\times {\hat{\sigma }}_{j}},\;j=1,2,\ldots ,k$$

## 3. Interviews Responses Processing and Results

To have a clear overview of the students’ perceptions in the case of hybrid educational systems, this section provides a step-by-step analysis according to GT (see Figure 1) having as input the responses collected based on the research questions from the Appendix A. The outcome of this analysis is a theoretical model for the Romanian higher education system in the COVID-19 pandemic situation which comes after the interview responses processing and the analysis of their connection with the relevant related works (see the literature sorting step from Figure 1).

### 3.1. Data Processing Based on GT Approach

As described in the Methodology section, the first step of our analysis consists of the calculation of the sample size which has high importance in the description of respondents’ distribution. After calculation, a sample size of *n* = 149 students (number of interviews) was obtained, 75 male (50.3%) and 74 female (49.7%). Interview respondents have ages ranging from 18 to 26 years. The student distribution *n*_1_, *n*_2_, and *n*_3_ are presented in Table 1.

The 149 interviews have been completed over a one-month period from the 18th of December 2020 when the initial information took place till the 14th of January. The respondents have been enrolled in one of the three faculties that have adopted the hybrid approach in Politehnica University Timisoara for the first four weeks of the first semester in 2020–2021. With a total number of 668 enrolled students in the first year of studies for the three faculties, the 149 interviews are representative. Even if Politehnica University Timisoara has a total of ten faculties, only the three already stated have adopted the hybrid approach, the rest started directly in an online system.

The data coding process was a constant comparison between the theory and theoretically sampling interview cases. Data from interview notes and transcriptions have been conceptualized line by line, while the iterative phenomena in the text have been temporary labeled. The coding process started from open coding—where labels have been offered to different ideas, grouping concepts at an abstract level—axial coding—whereby the main categories have been formed—and selective coding—where the categories have been integrated to form the initial theoretical framework.

The analysis started with the chronologic order of the interview data arranged in a memo type form and the retrieved codes have been numbered sequentially, adding new codes with each interview data analysis based on constant comparison of data. Clusters of initial codes have been formed till basic categories emerged. The open coding phase comprised a total number of 149 memos which contain 220 new original concepts. Codes were raised at a conceptual preliminary category level. The constant comparison process continued until all possible categories have been concluded. The axial coding phase comprises 13 categories (Table 2) based on 203 of the concepts retrieved from the open coding phase, 3 concepts have been encountered as similar in formulation and 14 concepts have been abandoned due to lack of relevance for the present study.

**Figure 2:**
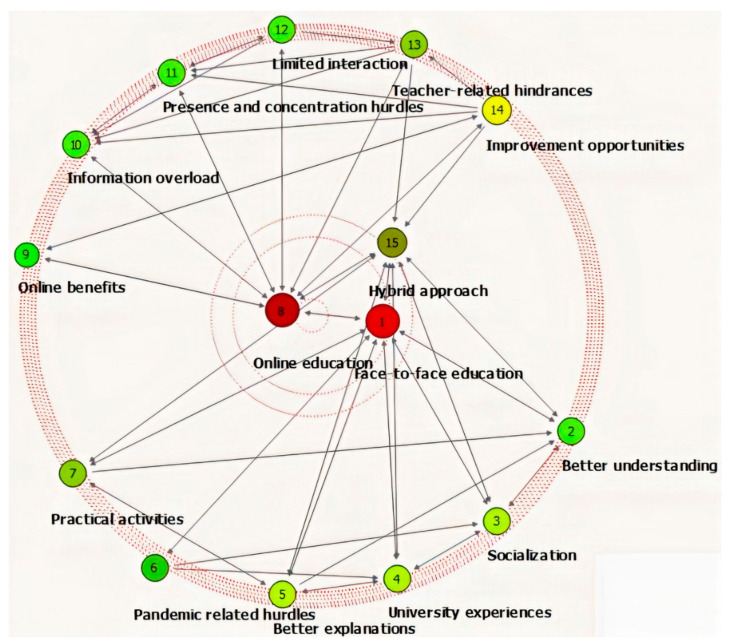
Relationships between retrieved categories.

The selective coding process explored the relations between categories in the search of core categories to which most codes will furthermore relate to. The 13 categories have been related to face-to-face education and online education, these two concepts being the focus of the present article. The open-access Social Networking Visualizer (SocNet V) (Patras, Greece), tool has been used because it enables a visual understanding of the strong relationships between the 13 formed categories and the two envisaged educational systems (Figure 2).

Starting from the idea of centrality in a graph-theoretic network, Grofman and Owen [25] hypothesis has been that the more central a position in a network, the more likely is the occupant of that position to emerge as a leader. For the relevancy of the relationship network, a degree of centrality (DC) has been computed for the 15 nodes (13 categories and two proposed central nodes: face-to-face and online education) and it is presented in Table 3. DC index in directed networks (as the one in our case) is the sum of outbound arcs from node u to all adjacent nodes. DC’ is the standardized index that can be computed taking into consideration DC divided by N-1 (non-value-added nets) or by sum DC (valued nets). DC range: 0 ≤ DC ≤ 14 and DC’ range: 0 ≤ DC’ ≤ 1.

As we can see from Table 3, the three educational systems, namely face-to-face, hybrid and online represent the most important links (leaders) to the network. Face-to-face education is central for better understanding, socialization, university experience, better explanations, pandemic-related restrictions and practical activities and online education is the core category for online benefits, information overload, presence and concentration hurdles, limited interaction, teacher-related hindrances, and improvement opportunities. The hybrid approach connects the two main core categories being associated with specific characteristics like adaptation, improvement, transition, and better accommodation.

The study of the grounded theory approach is continued with a second theoretical sampling stage named literature sorting, where researchers compare the emergent theory to the existing literature to model the results in a theoretical manner.

### 3.2. Literature Sorting, Discussions and Final Theoretical Model Proposal

Thornberg defines bibliographic analysis as a constructive method of GT [21], which gives liberty to the researcher without forcing data. Literature sorting is left until this moment for not jeopardizing data from the start with preconceived ideas [26].

In the last decades and especially in the last year with the coronavirus outbreak, scholars have been concerned with the study of differences between face-to-face, online, and blended or hybrid learning [27,28,29,30,31]. Even if [32] was one of the most supportive articles of distance education, arguing that there is no difference between face-to-face and distance systems, [33] cited errors in the previous study. From their perspective, several critical factors differentiate face-to-face and online learning like the task, student and instructor characteristics and student motivation. The researchers from [27] found that students expressed less satisfaction in online learning than face-to-face learning in the following areas: instructor explanations, enthusiasm, openness and interest in student learning and course discussions, quality of questions, evaluation, and grading. Nevertheless, Soffer and Nachmias [28] consider that online courses are as effective as, or more effective than, face-to-face courses.

The present study on Romanian first-year students’ perceptions presents a mixed result in this line of reasoning. There is a clear preference for face-to-face learning, probably due to a better understanding of information, tasks, and evaluation criteria [29]. The students involved in the current study emphasized that face-to-face education helps them understand and be attentive throughout the class (62 explicit answers that linked face-to-face education with better understanding). Thus, feedback is immediate, the information is easier to remember and they have more courage to ask questions or say that they did not understand something.

More than 85% of interviewees expressed that they want to go back to school and interact face-to-face with one another. This is correlated with [27] the argument that the lack of face-to-face interaction can leave students feeling isolated. Also, it strongly links face-to-face education with socialization needs (34 students explicitly linked face-to-face education with better interaction). Other scholars militating for socialization in learning environments are [34] who researched student experience in university transitions and [35] who studied student engagement with information technology. The latter also linked face-to-face socialization with university experiences, by saying that “supportive campus environment is a measure of the degree to which students’ perceive that their institution supports their academic and social needs and the quality of relations among different groups on campus, including students, faculty, and administrators”. Throughout our research university experiences have been often associated with student dorm room socialization, life on your own, friendships and student guidance in learning.

If face-to-face is influenced and influences student interactions or university experiences, this traditional education system is also linked with better teaching or better explanations as argued by [27,30,36]. This situation is not new. Indeed, [36] considers that the tendency to shift the conversation to traditional environments where we are more comfortable is normal since it is closer to our pedagogical beliefs. Interviewed students opinioned that their teachers are well trained for both face-to-face and online teaching but in traditional education environment they exert higher impact due to nonverbal communication feedback.

Practical activities can be a reason for the success and longevity of face-to-face learning. [37] raises the question of whether online teaching encourages creative thinking. The authors of [38] argues that the effectiveness of teaching and learning in engineering disciplines is strongly determined by practical exercises, experiments, and laboratory classes. Our interviewees connected practical activities with better understanding and better explanations, being eager to make experiments once again in the laboratory.

Besides the advantages of face-to-face education, there have been hurdles due to pandemic restrictions that impacted learning processes. As argued by [39], although undoubtedly essential in protecting participants’ health, there are numerous physiological, psychological, social, and economic complications associated with the wearing of masks. Socialization in face-to-face learning is considerably hindered by wearing masks. Some of the discussions with the sample size student group concluded that it was nice to meet colleagues even with masks, but it was not the same thing. COVID-19 has an impact on students undergoing physical distancing as worries for their health that hinders them to carry out daily activities [40]. Also, Fraser at al. conclude that the limitation of physical distance will determine a variety of mental and psychological problems [41]. Our students considered that distance restrictions have impacted their socialization needs and their university experiences.

Even if interviewees preferred face-to-face learning, they also considered as necessary to use the university online platform even after the pandemic and saw as possible (and even desire that) university courses to be performed in a blended manner. Thus, the hybrid approach brought a different mindset and new challenges to the educational system.

The hybrid system was considered by our students as protective, helpful for student life idea formation, a tool for a gradual transition from traditional education to the online system. Students proposed improvements especially to the itinerary issues in this system to avoid close face-to-face and online shifting activities in one- or two-hours period. But in general, the hybrid system has implications on both face-to-face and online education, also on several of their characteristics.

Hybrid education started early with the emergence of technology-enabling environments. However, drop off rates were then as high as 70–90% as argued by [42] due to the poor quality of student experience [43]. The year 2020 with all its challenges due to the COVID-19 pandemic has clarified that it is time for emerging research directions, clarification of methodologies and practice communities because higher education has never been more important [44]. In some cases, hybrid education creates confusion because of different implementation strategies chosen by universities like the rotating presences in the classes [45]. Moreover, Santos et al. argue that the hybrid educational system mostly fosters a perception of safety when students are in the faculty [45]. According to [46], the teachers shall use the face-to-face interaction at the beginning of the hybrid learning period to provide clear instructions to students about the virtual tools, where to get help, and how the evaluation will be done [46]. The hybrid approach or sometimes called blended learning has been widely considered a solution for the current situation as a bridge towards distance learning adaptation at a global scale. For post-COVID-19 higher education strategies the hybrid approach can be improved to become an alternative to a changing world. Despite its adaptability to pandemic situations, hybrid learning has as the main challenge the need of support for enabling the implementation of technology-based and pedagogy-informed teaching [47]. In this direction, the development of new online educational platforms shall consider also a future possible pandemic situation when arising the need for online or hybrid learning. From Romanian students’ perception analysis results that they feel satisfied if an online educational platform provides functionalities as multiple devices availability, online access to scholar situation, online administrative documents/requests evidence and submission, chat services between the users etc. [48]. On the one hand, online education has been associated with inherent benefits, but on the other, with significant hurdles. If we study the benefits of learning online, some of the most important ones are time and cost efficiency [13,49,50,51]. Other stated benefits are flexible access to content and instruction at any time [13,52,53,54], from any place, and increase the availability of learning experiences for learners who are marginalized [50], cannot, or who choose not to attend onsite offerings. At the same time, low achieving and high achieving students benefit from more attention or personalized task allocation [55]. The present research results show that online benefits experienced by engineering Romanian students confirm the existing literature and add some more like increased course presence due to the ease in connection, information disseminated through a practical online educational platform and increased comfort. Regarding the educational platform, our interviewees proposed a list of improvements that could make the online experience even more attractive.

Interviewed first-year Romanian students expressed four main problems in online education, namely: information overload, limited interaction, teacher-related hindrances and presence and concentration hurdles. Among the difficulties encountered in the online environment by scholars, [56] presents technical issues as most important, followed by teachers’ lack of technical skills, teaching styles improperly adapted to the online system and the lack of interaction with teachers or peers. 

Teacher-related hindrances cover up a wide pallet of issues. Firstly, many teachers have not been previously familiar with online platforms [57]. Thus, they just translate their discipline for the online environment instead of adapting it. Secondly, interviewees expressed feelings of demotivation when the teacher was reading from a PowerPoint presentation or when explanations have not been adequately provided for difficult tasks. In most cases, they consider that teachers have very high expectations without providing appropriate course instructions.

Regarding the limited interaction of the online environment, [58] and [59] argued that the lack of physical presence of classmates determines feelings of isolation. This lack of communication and interaction with teachers and peers is considered by [60] as the main challenge students face in online learning. The difficulty to socialize and ask questions has been well expressed by our students.

Information overload has been addressed by the Organization for Economic Cooperation and Development [61] when discussing the challenges in the online environment, namely, keeping an equilibrium between online courses to avoid students spending many hours in front of a screen that could affect their health. Our students also complained about the difficulty to understand a course and to solve a task, linking information overload with teacher-related hindrances. Student motivation, presence and drop out incentives are linked with information overload in online environments.

The presence and concentration hurdles are the most addressed difficulties in online education by our sample group. 43 from 149 students explicitly linked online learning with serious concentration hurdles. Most of them consider online presence a false presence since they are not attentive to the teacher as in face-to-face environments. The lack of concentration is put on account of family, phone, perceived easiness, or other priorities’ distractions. The authors from [56] gives them justice by saying that it is more difficult to study and be focused online. They also consider technical issues to hinder their online presence like poor internet connections, power failures or educational online platform blockage. The research from [59] emphasizes that during the university closure and lockdown, students may be developing feelings of fear, stress, and worry due to internet problems. Capone et al. conducted a research in the field of student mental well-being and the analysis has shown that the information-seeking level during the pandemic increase in the direction of collective risks, personal risks and psychological well-being etc. [62]. The study from [63] also suggests that technical availability is the core characteristic of successful online education. If we do not provide this availability to all students, presence and even concentration hurdles may often occur.

In the end, interviewees have proposed a set of improvements for both hybrid and online education. Regarding teacher-related hindrances, they consider as very important that teachers should adapt their discipline to the hybrid or online environment. The study conducted on [56] expresses the same idea by saying that online obstacles can be overcome with the help of teacher adaptability. The authors of [59] argues that teachers themselves will be subject to significant learning both in a move to different pedagogic approaches as well as needing to become expert users in the technologies employed. Garcia-Alberti et al. [64] conclude that there is much room for improvement, especially when focusing on the formative assessment.

As suggested by some of our sample group, students’ hindrances can also be overcome if students become responsible, understand to differentiate between learning time and family time/space for concentration purposes, and start discovering ways to synthesize information and manage their time efficiently and effectively. In the end, virtual platform improvements are also suggested.

**Figure 3:**
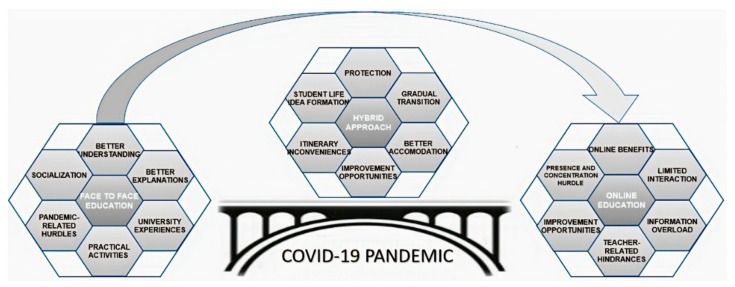
A theoretical model for the Romanian higher education system in the COVID-19 pandemic situation based on student perception.

The responses of our first-year students determined a theoretical model which visually expresses the current Romanian higher education situation in engineering specialities. In Figure 3, the proposed theoretical model is outlined.

The hybrid approach is associated with all its perceived characteristics and is presented in the COVID-19 lockdown situation as a necessary bridge from exclusive face-to-face to online education. Even if face-to-face education is preferred, in some regions with a high number of COVID-19 cases, the hybrid system will be a solution. But lessons learned from a first hybrid semester in Politehnica University Timisoara, Romania need to be applied for improvement purposes for semesters to come. In addition, when the pandemic will end, blended learning can continue in adaptable forms for student satisfaction and qualitative results. In the future, if higher education institutions gain sufficient experience and open to students’ needs, it will be possible to offer them the choice between various forms of teaching without being constrained by reasons of a health emergency.

## 4. Conclusions

This paper analyzed the experience of first-year students with the Romanian hybrid university educational system. The COVID-19 pandemic forced the movement of the educational process to an online form in the middle of March 2020. This had a real psychological impact on both students and teachers that had to adapt faster to this type of education.

The period before the beginning of the new university year 2020–2021 was full of uncertainties regarding the evolution of COVID-19 cases. This brought also uncertainty regarding how the universities shall proceed. Politehnica University of Timisoara was one of the Romanian universities that decided to implement the hybrid educational system for the first year students from three faculties. These students had face-to-face practical activities (laboratories, seminars) and the courses continued in an online manner.

Online asynchronous interviews were performed after the hybrid educational period ended. This type of interview was preferred due to its compliance with the COVID-19 restrictions. The results were analyzed by applying the GT approach.

The results of this research confirmed previous studies related to face-to-face and online education. The main outcome of this research is the theoretical model regarding the hybrid education system obtained based on student perception because this hybrid system was not so much researched in the past. Moreover, the literature sorting step of the GT approach considered the newest research related to hybrid education phenomena in the context of the COVID-19 pandemic. These studies focus on the students’ attitude changes during the teaching process, their forced adaptation to full-online or hybrid education because of the pandemic restriction, the psychological impact related to the physical distancing protective measures, concentration issues during courses, and practical activities, etc. Moreover, the proposed model incorporates the changes and the restrictions specific to pandemic situations.

Besides this important outcome, there are still some disadvantages to address. This research addressed the specific Romanian education system, and the theoretical model is available for this case. Further works need to be done to obtain a general theoretical model for hybrid education systems. In this regard, this study shall be applied in several universities around the world and based on them the identified characteristics can be classified in local (university-/country-specific) characteristics, or in a general category that is applicable worldwide.

## Figures and Tables

**Table 1:** Student distribution.

Layers	Faculty	Number of Respondents	Percentage (%)
*n*_1_	Industrial Chemistry and Environmental Engineering	27	18.1
*n*_2_	Mechanical Engineering	46	30.8
*n*_3_	Management in Production and Transportation	76	51.1
Total		149	100

**Table 2:** The 13 categories formed in the axial coding phase of the research.

No.	Category Name	Main Codes Describing the Category
1	Better understanding	Easy to remember, channelled concentration, 100% attention, attention due to respect, immediate feedback, open and free environment, courage to speak
2	Socialization	Better interaction, friendships, the importance of interaction, meeting colleagues psychically important, openness towards colleagues, profound interactions, a warm welcome, teamwork
3	University experiences	University reputation, discovering interesting faculty elements, university library at hand, student unity/guidance (student dormitory), student dorm room—life on your own
4	Better explanations	Trained teachers, clarifications, blackboard explanations, examples, more student feedback for teachers, higher teacher impact on students, the teacher observes non-verbal communication, pleasure to teach
5	Hurdles due to pandemic restrictions	Distance restrictions, restrictions to gather/discuss in a high number, higher cost but it is worth it, know colleagues even with masks
6	Practical activities	Laboratory understanding, focus on practical aspects, great to do yourself the experiments
7	Online benefits	Time savings, better presence, easy to connect, half course time, comfortable, online experience in high school helpful, online useful for documentation, Virtual Campus practical for grade/course/tasks visualization, online advantage—information at your fingertips
8	Information overload	Partial understanding, difficulty, no incentives, low motivation to learn, not efficient, online not helpful, low productivity, difficult to prepare for tasks/exams online, the online reason for dropout, large quantity of homework/tasks, long time to solve tasks/homework, information loss, online difficulty perceiving information details
9	Presence and concentration hurdles	False presence, stress due to power shortage risk/internet connection problems, low online presence due to lightness, tiring in front of a screen, vision destruction, home commodity not productive, concentration hurdle, easy to distract, concentration hurdle—family, friends, phone, not so seriously taken
10	Limited interaction	Difficulty to know colleagues/teachers, no student questions, difficult to speak/respond more than one student, students used to socialize online but not for deep relationships
11	Teacher-related hindrances	Teacher re-evaluation, teachers demotivated by student lack of attention, large information quantity in half time, high teacher demands and low offering, course structure not adapted/fitted to online teaching teachers do not upload courses on the Virtual Campus, teachers that do not open their camera—disengaged students
12	Improvement opportunities	The teacher demands adapted for online education, being autodidact (self-taught), time management, online listening habits need to change, responsible student, challenge to use more virtual platforms, summaries/conspectus for lessons to understand, Virtual Campus problems (connection errors, difficulty in accessing some courses), CV improvement proposition (background personalization, time limit removed/time loading changed, grades/presence visualization per student, the organization for mobile use)
13	Hybrid approach characteristics	Transition/adaptation towards online learning, if not face-to-face, hybrid preference, hybrid system helpful, itinerary problems, hybrid system- student life idea formation, better accommodation to faculty, hybrid optimization, face-to-face explanations about the online, hybrid system—a gradual transition, hybrid approach protects students, student’s wellbeing the priority, hybrid approach helpful to familiarize with town/university/facilities/colleagues/teachers

**Table 3:** The degree of centrality for the 15 nodes network.

Node	Category	DC	DC’	%DC’
1	Face-to-face education	8.000	0.571	57.1
2	Better understanding	3.000	0.214	21.4
3	Socialization	4.000	0.285	28.5
4	University experience	4.000	0.285	28.5
5	Better explanations	5.000	0.357	35.7
6	Pandemic-related restrictions	3.000	0.214	21.4
7	Practical activities	4.000	0.285	28.5
8	Online education	8.000	0.571	57.1
9	Online benefits	2.000	0.142	14.2
10	Information overload	2.000	0.142	14.2
11	Presence and concentration hurdles	3.000	0.214	21.4
12	Limited interaction	4.000	0.285	28.5
13	Teacher-related hindrances	4.000	0.285	28.5
14	Improvement opportunities	6.000	0.428	42.8
15	Hybrid approach	7.000	0.500	50.0

## Data Availability

Data available on request due to ethical restrictions.

## Appendix A

**Table A1:** In-depth interview for determining student’s satisfaction level regarding the hybrid education system in Politehnica University Timisoara—Interview Guide.

1.	What have been your expectations regarding your faculty/university before starting classes?
2.	Has it been beneficial for you to start the seminars and laboratories in a face-to-face system? If yes, in what way has it helped you? Would you repeat the experience in the current pandemic situation?
3.	What was the impact of the online system after the first month of hybrid education? Has it helped you coming to the faculty to understand/accommodate with online courses? The transition towards online education has been easier in this way?
4.	Please detail your interactions with colleagues and teachers in both systems: face-to-face and online. Are these social interactions important for you/your learning activity?
5.	Have you opted for student dorm rooms? If yes, what was the influence of this ac-commodation on your interpersonal relationships’ development opportunities and your learning process?
6.	Are teachers prepared to teach online? Have they adapted their discipline materi-als/content? In which of the two systems have they better explained the con-cepts/problems?
7.	How interactive, appealing and practical is the Virtual Campus (specific online edu-cational platform for UPT)? What improvements would you suggest for its optimiza-tion?
8.	Subjects are easier to retain/understand in face-to-face or online systems? Tasks/homework have been more/harder in the online or traditional system?
9.	Is the structure of the courses appropriate for both systems (face-to-face and online)? Mention (if it is the case) what should be added, changed or adapted, in your opinion.
10.	Do you learn better face-to-face or online? Have you changed recently the way in which you listen/learn? Can you concentrate in the online environment better or the same as in the traditional version? What are the challenges the online education brought in this sense?
11.	Your presence has improved in the online environment? Is it easier/convenient this way? Do you save time compared to the traditional face-to-face presence?
12.	The hybrid system is adequate for the current situation? Do you see it possible for the next year? Or do you prefer exclusive face-to-face or exclusive online education?
13.	Age:
14.	Gender:
15.	Faculty:
16.	Specialty:
